# Contrast-Enhanced Voiding Urosonography in Pediatric Vescicoureteral Reflux: A Narrative Review

**DOI:** 10.3390/diagnostics16213531

**Published:** 2026-10-27

**Authors:** Anna Russo, Lucrezia Bucciero, Vittorio Patanè, Stefano Guarino, Anna Di Sessa, Pierluigi Marzuillo, Alfonso Reginelli

**Affiliations:** 1 Precision Medicine Department, University of Campania “Luigi Vanvitelli”, 80138 Naples, Italy; 2 Department of Woman, Child and of General and Specialized Surgery, University of Campania “Luigi Vanvitelli”, 80138 Naples, Italypierluigi.marzuillo@unicampania.it (P.M.)

**Keywords:** contrast-enhanced voiding urosonography, vesicoureteral reflux, cystosonography, pediatric ultrasound, intrarenal reflux, voiding cystourethrography, ultrasound contrast agents, urinary tract infection

## Abstract

Contrast-enhanced voiding urosonography (ceVUS) is a radiation-free technique for evaluating pediatric vesicoureteral reflux (VUR), although urethral catheterization is required. This narrative review summarizes its technical principles, diagnostic performance, safety, limitations, and clinical applications. Comparative studies suggest broadly similar diagnostic performance between ceVUS, voiding cystourethrography (VCUG), and direct radionuclide cystography; some report higher detection rates or reflux grading with ceVUS, but formal equivalence or superiority has not been established. Continuous real-time observation and repeated filling–voiding cycles may facilitate detection of intermittent reflux and intrarenal reflux, whereas grade I reflux confined to a non-dilated distal ureter may be missed. ceVUS-detected intrarenal reflux has shown a topographic association with acute cortical defects on dimercaptosuccinic acid scintigraphy, although its prognostic significance remains uncertain. Beyond initial diagnosis, ceVUS has been used for follow-up after conservative or surgical treatment, renal transplantation, and radiation-free videourodynamics. Intravesical second-generation microbubble agents have shown favorable short-term safety and parental acceptance. Nevertheless, evidence is limited by heterogeneous protocols, operator dependence, imperfect reference standards, and scarce data on clinical outcomes. Overall, ceVUS is a promising radiation-free option for selected pediatric VUR pathways, while fluoroscopic imaging remains appropriate when detailed urethral or complex distal ureteral anatomy is required.

## 1. Introduction

Vesicoureteral reflux (VUR) is defined as retrograde passage of urine from the urinary bladder into the ureter and, in more advanced cases, the renal collecting system [1,2]. Its clinical relevance is not determined by reflux alone but by its interaction with urinary tract infection (UTI), bladder dysfunction, congenital urinary tract abnormalities, and susceptibility to renal parenchymal injury [3]. The traditional imaging reference has been fluoroscopic voiding cystourethrography (VCUG), which combines dynamic assessment with standardized five-grade anatomical classification [4,5,6]. However, VCUG requires urethral catheterization, exposes children to ionizing radiation, and samples an intermittent phenomenon using intermittent fluoroscopic acquisition [7,8].

Beyond conventional grayscale ultrasonography, contrast-enhanced ultrasound (CEUS) has been increasingly investigated in genitourinary imaging for the characterization of solid renal masses [9,10], complex renal cysts [11], and bladder tumors [12], primarily through the intravenous administration of microbubble contrast agents to assess tissue vascularization and enhancement patterns [9,10,11,12].

In contrast, contrast-enhanced voiding urosonography (ceVUS), also described as contrast-enhanced cystosonography or voiding urosonography, represents a distinct intracavitary application that uses intravesically administered microbubbles and contrast-specific low-mechanical-index ultrasound to visualize vesicoureteral reflux in real time [13,14]. Prospective and retrospective studies have compared ceVUS with VCUG, direct radionuclide cystography, surgical findings, and renal cortical scintigraphy, while newer reports have evaluated ceVUS in follow-up protocols, kidney transplantation, and videourodynamics [15,16,17,18,19,20,21,22,23,24,25,26,27,28,29,30,31,32].

## 2. Methods

This review was developed as a targeted narrative synthesis of original studies addressing pediatric contrast-enhanced voiding urosonography (ceVUS) for the diagnosis and assessment of vesicoureteral reflux (VUR). A targeted literature search was performed to identify studies evaluating the diagnostic performance, technical aspects, clinical applications, and safety of ceVUS, with particular attention to comparisons with established reference techniques.

Literature searches were conducted in PubMed (MEDLINE), Scopus, and Web of Science, with the final search performed on the 31 August 2026. The database-specific search strategies, including the exact Boolean syntax and applicable search restrictions, are provided in Supplementary Table S1. The search strategies included terms related to contrast-enhanced voiding urosonography and its commonly used synonyms (“contrast-enhanced voiding urosonography”, “ceVUS”, “voiding urosonography”, and “contrast-enhanced cystosonography”), vesicoureteral and intrarenal reflux (“vesicoureteral reflux”, “VUR”, and “intrarenal reflux”), and pediatric populations (“child”, “children”, “pediatric”, “infant”, and “adolescent”). These terms were combined according to the database-specific Boolean equations reported in Supplementary Table S1. Comparator modalities, including voiding cystourethrography (VCUG), direct radionuclide cystography, and dimercaptosuccinic acid (DMSA) scintigraphy, were considered during study selection and narrative synthesis but were not explicitly included in the Boolean search strings.

Priority was given to paired comparative studies against VCUG or direct radionuclide cystography, studies evaluating inter-reader reliability or the relationship between VUR detected by ceVUS and renal cortical abnormalities, and clinical cohorts addressing follow-up, safety, transplantation, or urodynamic applications. No separate database searches specifically targeting safety or technical applications were conducted. Studies addressing these topics were identified among the records retrieved through the main search strategy and were considered eligible when relevant to the objectives of the narrative review.

Veterinary studies and adult-only experimental studies were not included in the principal synthesis. Because the review was conceived as a narrative rather than a systematic evidence synthesis, study identification and selection were not conducted according to a formal PRISMA workflow, and record-level screening counts were not prospectively collected.

To support a transparent and critical interpretation of the available evidence, the principal comparative and clinical follow-up studies underwent a structured qualitative methodological appraisal (Table 1).

Five predefined domains were considered: patient selection, blinding and independence of interpretation, appropriateness of the comparator or reference standard, completeness of verification, and comparability of imaging protocols and examination sequence. Each domain was classified as having low, high, or unclear methodological concern based on the information reported in the original publications. Low concern was assigned when adequate methodological safeguards were documented; high concern was assigned when identifiable methodological limitations could materially affect the interpretation of findings; and unclear concern was assigned when insufficient information was available to support either judgment. Brief study-specific justifications were provided to support the assessments. This qualitative appraisal was intended to facilitate critical interpretation within the framework of a narrative review and did not constitute a formal QUADAS-2 assessment or an aggregate methodological quality score.

A narrative approach was considered necessary because of the marked variability of the available literature and the heterogeneity of the reported data, including differences in study design, patient populations, ultrasound contrast agents, bladder-filling protocols, units of analysis, VUR grading systems, and reference standards. This heterogeneity limits direct comparability across individual studies and requires caution when interpreting pooled estimates. However, quantitative syntheses of ceVUS diagnostic performance have been reported in previous systematic reviews and meta-analyses. The present work was intentionally designed as a narrative review rather than a de novo quantitative synthesis, in order to integrate diagnostic performance with technical considerations, reflux grading, intrarenal reflux, safety, follow-up applications, transplantation, and videourodynamic use.

## 3. Discussion

### 3.1. Technique, Contrast Agents, and Protocol Determinants

CeVUS begins with baseline grayscale ultrasonography of the kidneys, collecting systems, ureters when visible, and bladder. After aseptic transurethral catheterization, the bladder is emptied to reduce dilution and layering artifacts. A second-generation microbubble agent is then administered intravesically, usually diluted in normal saline, followed by slow bladder filling. No fixed post-contrast delay is required before ultrasound assessment. Contrast-specific imaging should begin as the contrast-containing solution enters the bladder and should continue throughout bladder filling, because microbubbles become sonographically detectable within seconds and reflux may occur transiently at any stage of the filling–voiding cycle. The bladder, distal ureters, and kidneys should therefore be assessed continuously or in rapid alternation during filling, at maximal tolerated bladder capacity, during voiding, and immediately after voiding. Prolonged delays are undesirable because progressive microbubble destruction or heterogeneous intravesical distribution may reduce contrast conspicuity. The examination is performed with contrast-specific software at a low mechanical index, generally below 0.10–0.20, to limit premature microbubble destruction. The bladder and kidneys are assessed repeatedly during filling, at maximal tolerated filling, during voiding, and after voiding. When urethral assessment is required, transperineal, translabial, or interscrotal imaging is added during micturition.

Although ceVUS avoids ionizing radiation, it remains an invasive examination because transurethral bladder catheterization is required, similarly to conventional VCUG. Catheterization may cause discomfort and may contribute to transient dysuria, minor bleeding, or urinary tract infection; therefore, the radiation-free nature of ceVUS should not be equated with a non-invasive procedure.

In their prospective single-center experience, Tamasi et al. reported an examination duration of approximately 15–30 min [33]. However, actual examination time may vary depending on bladder-filling time, patient cooperation, the number of filling–voiding cycles performed, and the need for dedicated urethral imaging.

Sulfur hexafluoride lipid-type A microspheres, marketed as SonoVue or Lumason, are the most frequently reported agent in the contemporary pediatric literature. Unlike pediatric CEUS examinations of the kidneys and adrenal glands, which generally rely on intravenous microbubble administration [34], ceVUS involves intravesical contrast administration and therefore requires a distinct examination protocol.

Contrast-agent administration protocols vary across studies. For SonoVue, Kis et al. administered 1 mL intravesically, whereas Kim et al. used 0.3 mL as part of the intravesical contrast mixture during simultaneous ceVUS and VCUG [14,18]. Optison, which contains octafluoropropane microbubbles within a human serum albumin shell, has also been evaluated prospectively and retrospectively. Ntoulia et al. reported a mean dose of approximately 0.3 mL in a prospective comparison with VCUG, while Paltiel et al. reported a median dose of 0.15 mL in a larger retrospective accuracy study [15,19]. These studies support the technical feasibility of non-sulfur-hexafluoride agents, although their intravesical use may be off-label depending on jurisdiction.

Repeated filling–voiding cycles are important because VUR is intermittent. Cyclic ceVUS can detect reflux that is absent during the first cycle without adding radiation, and the second cycle may require only saline if sufficient residual microbubbles remain. Several comparative studies used two cycles routinely [16,17,21], whereas other protocols used one cycle for each modality [19]. The number of cycles, therefore, is not a trivial technical detail: it directly changes the probability of observing transient low-volume reflux.

Bladder filling volume is another major determinant. Excessive filling may provoke or exaggerate reflux, while insufficient filling may fail to reproduce clinically relevant bladder pressure. Viljamaa et al. specifically limited filling to age-adjusted expected bladder capacity and found comparable patient-level VUR detection between ceVUS and direct radionuclide cystography [21]. By contrast, the Wang et al. protocol used more than twice the predicted bladder volume, a choice that complicates interpretation of the higher ceVUS detection rate because overdistension may induce iatrogenic reflux [22]. Physiologic filling, documentation of the volume at first reflux, and clear reporting of the pressure or filling endpoint should become standard elements of future studies.

Contrast distribution must remain homogeneous. Residual urine may cause microbubble layering and obscure the bladder neck, as described during ceVUS urodynamics [32]. Prior iodinated contrast can also interfere with subsequent ceVUS if both studies are performed sequentially. The sequence of paired examinations therefore matters. Some studies performed VCUG first [15], others ceVUS first [17,20,21,22], and Kim et al. avoided sequence-related biological variability by performing both examinations simultaneously with a combined intravesical mixture [18]. The simultaneous design is methodologically attractive for an intermittent phenomenon, but the mixed contrast solution does not reproduce routine independent performance of either modality.

### 3.2. Diagnostic Performance Compared with Conventional Reflux Imaging

Throughout this section, sensitivity, specificity, predictive values, and accuracy are considered measures of diagnostic performance when an explicit reference standard is designated, whereas percentage agreement and kappa statistics are treated as measures of concordance or reproducibility and not as measures of diagnostic accuracy. Ninety-five percent confidence intervals are reported when provided in the original publications. The principal comparative studies evaluating ceVUS against VCUG or direct radionuclide cystography are summarized in Table 2.

Across the comparative literature, ceVUS demonstrates diagnostic performance that is generally comparable to VCUG, but discordance is common enough to challenge the concept of VCUG as an infallible reference standard. In the prospective Optison trial by Ntoulia et al. [15], 30 children and 62 pelviureteric units underwent same-day ceVUS and VCUG. Using VCUG as the reference standard, ceVUS achieved a sensitivity of 91.7% (95% CI, 61.5–99.8%) and a specificity of 98.0% (95% CI, 89.4–99.9%). Separate concordance measures showed 84.3% agreement for reflux detection and 81.8% agreement for grading. The main discordances involved low-grade reflux, and no serious acute contrast-related adverse event was observed.

Mane et al. evaluated 30 children and 58 kidney–ureter units and reported 17 refluxing units on VCUG and 21 on ceVUS. Relative to VCUG, the reported sensitivity was 100%, specificity 87.8%, positive predictive value 77.3%, and negative predictive value 100%, with a Cohen kappa of 0.84 [16]. The study supports high concordance but contains internal numerical inconsistencies: the Results section reports 21 refluxing units detected by ceVUS and an overall specificity of 87.8%, whereas the Discussion reports 22 refluxing units and a specificity of 90.24%. Its conclusions should therefore be used qualitatively rather than as a source for precise pooled estimates.

In infants with mild prenatal hydronephrosis, Siomou et al. [17] found reflux in 18 of 60 infants by ceVUS and in 8 of 60 by VCUG. At the kidney–ureter-unit level, ceVUS detected 25 positive units and VCUG 12, with moderate intermodality agreement. Most refluxes missed by VCUG were grade II or III, whereas the single reflux missed by ceVUS was grade I. This study is clinically important because it demonstrates that discordance is not restricted to trivial reflux and because both techniques were performed cyclically in the same session. However, without an independent reference standard, the findings can indicate either VCUG underdetection, ceVUS overclassification, or both.

Kim et al. [18] offered one of the most methodologically informative designs by performing ceVUS and VCUG simultaneously in 32 children with 63 urinary systems. Twenty-seven systems had VUR. CeVUS identified 23 of the 27 refluxing systems and VCUG 26 of 27; no statistically significant difference in detection rate was identified (*p* = 0.370). However, this finding should not be interpreted as evidence of equivalence or non-inferiority, as the study was not designed for that purpose. All four refluxes missed by ceVUS were grade I, whereas the single reflux missed by VCUG was grade II. Intermodality agreement for VUR grading was very good (weighted κ = 0.89; 95% CI, 0.81–0.96). These findings indicate strong grading concordance between the two modalities in this cohort, while also highlighting the potential vulnerability of ceVUS for transient grade I reflux confined to a non-dilated distal ureter.

Paltiel et al. [19] retrospectively reviewed 101 paired Optison ceVUS–VCUG examinations in 97 children, with blinded independent re-reading of ceVUS by two experienced pediatric radiologists. Sensitivity ranged from 75.0% to 85.7% according to side, and inter-reader kappa for ceVUS grading was 0.76–0.78. The study is notable for blinded assessment and reproducibility analysis, although the sequential rather than simultaneous performance of ceVUS and VCUG may have influenced detection of an intermittent phenomenon such as VUR. It nevertheless provides evidence that diagnostic accuracy remains clinically useful even when stricter blinded reading is applied.

Jottrand et al. [20] prospectively studied 89 children and 192 pelviureteric units. Reflux was identified by at least one technique in 54 units, by ceVUS alone in 21, by VCUG alone in 3, and by both in 30. Among units positive with both modalities, ceVUS assigned a higher grade in 19 of 30, including reclassification from low-grade to high-grade reflux in 10. No unit was graded higher by VCUG. The authors explored an original physical explanation: at body temperature and high shear rate, the viscosity of the diluted ceVUS medium was almost identical to urine, while the iodinated VCUG medium was approximately six times more viscous. The hypothesis that lower viscosity facilitates more physiologic retrograde flow is plausible but remains associative because the study did not experimentally manipulate viscosity while holding all other imaging variables constant.

Viljamaa et al. [21] compared ceVUS with direct radionuclide cystography in a prospective blinded study of 22 children with recurrent UTIs and normal bladder function. CeVUS detected VUR in 77.3% of patients and 52.3% of renal units, while radionuclide cystography detected VUR in 72.7% and 50.0%, respectively. No statistically significant difference in patient-level detection was identified (exact McNemar *p* = 1.00), whereas renal-unit concordance was moderate (κ = 0.59; 95% CI, 0.35–0.83). All discordant findings involved non-dilating reflux. The study is especially relevant because filling was limited to physiologic capacity, reducing concern that the high sensitivity of ceVUS depends on bladder overdistension.

Wang et al. [22] reported a higher VUR detection rate with contrast-enhanced cystosonography than VCUG in 50 young infants after UTI. The study found 31 positive pelviureteric units by ceVUS and 18 by VCUG, with a kappa of 0.547. Interpretation requires caution because the source article contains internally conflicting denominators: it reports VUR in 27 of 100 pelviureteric units overall despite 31 units being positive with ceVUS, and its eligibility criteria state that VUR was confirmed by VCUG although only 9 of 50 infants were reported as VCUG-positive. In addition, bladder filling exceeded twice the predicted capacity. The diagnostic performance of ceVUS has also been evaluated in several quantitative syntheses. Chua et al. [35] reported pooled sensitivity and specificity of approximately 90% and 93%, respectively, in a meta-analysis of second-generation contrast-enhanced voiding urosonography, although substantial interstudy heterogeneity was present. A subsequent systematic review and meta-analysis including 36 studies similarly reported high overall diagnostic accuracy, with an area under the curve of approximately 0.97 and pooled sensitivity and specificity above 90% for second-generation contrast agents [36]. More recent meta-analytic studies have confirmed generally high diagnostic performance while emphasizing protocol heterogeneity, differences in reference standards, and risk of bias across the available literature [37]. Taken together, the comparative studies show substantial overlap in VUR detection and diagnostic-performance estimates between ceVUS and conventional reflux imaging. However, the available evidence does not establish formal equivalence or non-inferiority between these modalities. Apparent sensitivity and specificity depend strongly on which modality is designated the reference. Since reflux is intermittent and the tests are often sequential rather than simultaneous, a ceVUS-positive/VCUG-negative result cannot automatically be classified as a false positive. The most defensible synthesis is that ceVUS and VCUG sample the same dynamic disorder differently: ceVUS offers continuous observation and exquisite sensitivity to small numbers of microbubbles, whereas VCUG provides a wider anatomical overview and standardized radiographic documentation. Importantly, a higher detection rate or higher reflux grade with ceVUS should not, in itself, be interpreted as evidence of better clinical outcomes, reduced treatment requirements, or improved patient management.

### 3.3. Reflux Grading and Reproducibility

CeVUS grading mirrors the five-tier International Reflux Study system and is based on the level reached by microbubbles, ureteral and pelvicalyceal dilatation, calyceal blunting, loss of papillary impressions, and ureteral tortuosity. This parallel structure facilitates clinical translation from VCUG-based management pathways. Nevertheless, grading is not identical between modalities because ceVUS depicts microbubble distribution and renal morphology in real time, while VCUG reflects the concentration of iodinated contrast during intermittent fluoroscopy.

Reproducibility has been encouraging. Ntoulia et al. [15] reported more than 80% grading agreement between ceVUS and VCUG. Kim et al. [18] reported very good interobserver agreement for ceVUS grading (κ = 0.87; 95% CI, 0.82–0.92) and very good intermodality agreement between ceVUS and VCUG grades (weighted κ = 0.89; 95% CI, 0.81–0.96). Paltiel et al. [19] reported inter-reader kappa values of 0.76–0.78. Jottrand et al. [20] found interobserver kappa values ranging from 0.75 to 0.98 and intraobserver values from 0.93 to 0.98. These data suggest that ceVUS grading is reproducible in experienced hands, although most studies were performed in specialized pediatric centers.

The direction of discordance is clinically relevant. Several studies found a tendency for ceVUS to assign a higher grade than VCUG [17,20], whereas grade I reflux was more likely to be missed by ceVUS in the simultaneous Korean study [18]. This apparent paradox reflects two different limitations: ceVUS may detect greater proximal extension or IRR once reflux reaches the collecting system, but may miss a short-lived reflux confined to the non-dilated distal ureter. For clinical use, reporting should therefore document not only the final grade but also the phase of occurrence, the number of cycles, bladder volume at first detection, and the presence of intrarenal reflux.

### 3.4. Intrarenal Reflux and Renal Parenchymal Risk

Intrarenal reflux (IRR) is the extension of refluxed urine from the calyceal system into the collecting ducts and renal parenchyma. It is biologically important because infected reflux reaching susceptible papillae may contribute to acute pyelonephritis and subsequent scarring. Conventional fluoroscopy can depict IRR, but small or patchy extensions may be obscured by limited spatial resolution, contrast dilution, and dose-reduction strategies.

Kim et al. reported a higher frequency of IRR detection with ceVUS than with simultaneous VCUG. Among 27 refluxing urinary systems, 10 cases of IRR were identified with ceVUS and only 3 with simultaneous VCUG (*p* = 0.016). Although this paired finding supports greater IRR conspicuity with ceVUS in this cohort, it should not be interpreted as establishing the overall diagnostic superiority of ceVUS over VCUG. All IRR occurred with grade III–V reflux, and ceVUS depicted mild, multifocal, and extensive patterns that were not evident fluoroscopically [18]. Jottrand et al. similarly found nine cases of intraparenchymal reflux on ceVUS, all associated with grade V VUR, while VCUG showed none [20].

A large dedicated clinical series was reported by Cvitkovic-Roic et al. [38]. Among 5153 children undergoing ceVUS, VUR was identified in 1959 (38%), and IRR was detected in 233 of these children (11.9%). A total of 285 ureteral units showed IRR, with high-grade VUR (grades IV–V) accounting for the great majority of IRR-positive units. IRR was particularly associated with younger age, with grade IV VUR predominating among children younger than 12 months. The scale of this cohort supports an association between IRR, younger age, and severe reflux, although its retrospective single-center design and absence of systematic comparison with VCUG or DMSA limit causal interpretation. Oh et al. [26] evaluated whether IRR on ceVUS corresponds to acute renal cortical abnormalities on technetium-99m dimercaptosuccinic acid (DMSA) scintigraphy. In 27 infants and 54 renal units, ceVUS detected IRR in 8 units, while acute DMSA showed cortical photon defects in 10 units. Renal-unit concordance was 96.3%; relative to DMSA defects, ceVUS yielded 80% sensitivity, 100% specificity, and 96.3% accuracy. At lesion level, 15 of 19 photon defects corresponded to ceVUS-detected IRR, and every IRR focus identified by ceVUS had a matching DMSA abnormality. The four discordant lesions were all interpolar, highlighting the need for systematic transverse as well as longitudinal renal scanning.

These findings do not establish that ceVUS can replace DMSA scintigraphy. An acute DMSA defect reflects cortical dysfunction or inflammation, not IRR itself, and the two tests were not performed simultaneously. However, the close spatial association suggests that ceVUS provides more than reflux grading: it may identify the pathway by which infected urine reaches vulnerable parenchyma. Longitudinal studies comparing ceVUS-detected IRR with delayed DMSA scarring, differential renal function, recurrent febrile UTI, and treatment outcomes are needed before IRR can be integrated formally into management algorithms.

### 3.5. Clinical Applications Beyond Initial Diagnosis

Real-world cohorts support ceVUS across a broad spectrum of pediatric indications. Zhang et al. prospectively evaluated 90 children at high risk of VUR and found the examination feasible, diagnostically adequate, and free of reported adverse events. VUR was identified in 44 children and 65 of 178 pelviureteric units, including a substantial proportion of grade III–V disease. Rubelj et al. [23] reported their experience in 183 children, with 175 completed examinations and VUR in 38.9%. Reflux was significantly more frequent after recurrent UTI than after a first UTI, while age, sex, baseline urinary tract ultrasound, inflammatory markers, and causative organism were not reliable filters for VUR. These observations reinforce the limited ability of conventional ultrasound alone to exclude reflux.

Follow-up is arguably the most intuitive application of a radiation-free technique. The paired Marschner publications [24,25] describe a predominantly pediatric, although age-heterogeneous, cohort of 49 patients evaluated for suspected VUR, follow-up, or recurrence. No contrast-related adverse events were observed. In the management-focused report, 22 patients had reflux on ceVUS, 17 subsequently underwent surgery, and intraoperative findings confirmed reflux in all operated patients. The reported sensitivity of 95.7% and specificity of 100% must be interpreted cautiously because surgical verification was not applied uniformly and was concentrated among ceVUS-positive patients, producing verification bias. Nevertheless, the study illustrates that ceVUS findings can be integrated directly into treatment planning.

Tamasi et al. [33] evaluated a large prospective single-center follow-up strategy in 1050 children. Patients with grade III–V reflux underwent ceVUS reassessment at 12 months rather than waiting for conventional VCUG at 24 months. Among the reported follow-up cohort, substantial proportions showed complete resolution or downgrading, and antibiotic prophylaxis was discontinued in a subset according to the study-specific management protocol. These observations demonstrate that ceVUS findings were used to inform follow-up decisions in that cohort; however, they do not establish that ceVUS itself reduces antibiotic exposure, treatment burden, or adverse clinical outcomes. Moreover, major numerical inconsistencies exist between the number of patients eligible for follow-up, the number reported as completing follow-up, and the denominators used for antibiotic discontinuation. The study should therefore be interpreted primarily as evidence of the feasibility of a ceVUS-based follow-up strategy rather than as proof of treatment benefit.

In pediatric kidney transplantation, the superficial graft location creates an especially favorable acoustic window [39]. Cheng et al. retrospectively evaluated eight transplant recipients investigated with either VCUG or ceVUS. Four of five ceVUS patients had reflux, and imaging results informed timed voiding or ureteral reimplantation. No post-procedural infection or allergic reaction was reported, and local costs favored ceVUS [28]. Although the sample was too small for accuracy estimates, the study demonstrates a practical niche in which repeated radiation-free imaging is particularly attractive.

CeVUS is also suited to post-treatment surveillance after endoscopic injection or ureteral reimplantation because it can be repeated without cumulative radiation. These properties make ceVUS an attractive option for serial assessment in children with recurrent UTIs, congenital anomalies, solitary kidneys, or prolonged imaging follow-up. However, whether replacing or reducing VCUG examinations with ceVUS in these settings leads to equivalent or improved clinical outcomes, fewer interventions, or reduced treatment burden has not yet been established. Modality selection should therefore remain individualized according to the clinical question, particularly when detailed urethral or distal ureteral anatomy is required.

### 3.6. Safety, Tolerability and Parental Acceptance

Available studies generally support a favorable short-term safety profile for intravesical microbubble administration, although the strength of this evidence depends on the denominator, duration of observation, and method used to ascertain adverse events. In the largest dedicated prospective safety study, Papadopoulou et al. [40] evaluated 1010 children undergoing ceVUS with SonoVue. Adverse events were monitored during the examination and patients were followed by telephone for 7 days. No serious adverse event occurred; minor events were reported in 37/1010 children (3.66%), most commonly transient dysuria (26/1010, 2.57%). The investigators considered most reported symptoms likely related to urethral catheterization rather than to the contrast agent itself. Taken together, the available evidence supports a low observed rate of short-term adverse events after ceVUS, but the absence of events in small cohorts should not be interpreted as proof of the absence of rare complications. Furthermore, dysuria, urinary retention, minor bleeding, and post-procedural UTI may be related to urethral catheterization rather than specifically to intravesical microbubble exposure. Standard aseptic catheterization, screening for active UTI or known contrast hypersensitivity, appropriate observation, and explicit documentation of adverse-event ascertainment remain necessary.

Parental preference is clinically meaningful because many children require repeated reflux imaging. Avoidance of radiation is the dominant perceived benefit, but the ultrasound environment may also appear less intimidating than a fluoroscopy suite. In a prospective study, Seelbach et al. reported that 96% (54/56) of parents would agree to repeat ceVUS, while 92.9% (52/56) expressed a preference for ceVUS over conventional VCUG [27].

High acceptance should not, however, be used to minimize the discomfort of catheterization, which is common to both ceVUS and VCUG.

### 3.7. Limitations of ceVUS and Situation in Wich VCUG Remains Valuable

CeVUS is operator dependent. Continuous assessment requires the examiner to alternate rapidly between kidneys, ureters, bladder, and urethra while preserving the phase of filling or voiding in which reflux occurs. A transient event may be missed when the relevant structure is outside the field of view. This limitation is most evident for grade I reflux, which may be confined to a non-dilated distal ureter and may resemble adjacent bowel gas [18,20]. Operator training is therefore an important component of ceVUS implementation. However, no universally validated minimum number of examinations required to achieve proficiency has been established. In a dedicated learning-curve study performed at a center previously naïve to ceVUS, Velasquez et al. described an initial technical learning curve related primarily to contrast preparation and concentration, patient positioning, and optimization of ultrasound settings. After implementation of a standardized protocol, the authors reported that pediatric radiologists became comfortable performing the examination after observing and performing approximately two to four studies. This small single-center experience should not be interpreted as a formal competency threshold; supervised training, familiarity with pediatric urinary tract ultrasonography and contrast-specific imaging, and local assessment of technical and interpretative competency remain advisable [41].

Anatomical assessment of the distal ureter and ureterovesical junction is not consistently reliable. Benya et al. [29] reviewed 34 ceVUS studies with reflux and found clear visualization of the ureterovesical junction in only 9.6–23.1% of refluxing moieties and clear visualization of the distal ureter in 26.9–51.9%, depending on reader. Inter-reader agreement for clear ureterovesical junction visualization was poor. Duplication could be recognized with high inter-reader agreement when visible, but periureteral diverticula were not identified in the cohort. This limitation matters when surgical planning depends on insertion site, ectopia, periureteral diverticulum, or complex duplication.

Urethral assessment is feasible with optimized transperineal imaging, and several studies reported normal or concordant urethral findings in boys [17,22,32]. Nevertheless, fluoroscopic VCUG remains prudent when posterior urethral valves, urethral stricture, complex postoperative anatomy, or another obstructive urethral lesion is strongly suspected. The choice should be driven by the clinical question rather than by a universal replacement strategy.

Technical artifacts include premature microbubble destruction, excessive or insufficient contrast concentration, posterior acoustic shadowing from an intensely opacified bladder, separation of contrast from residual urine, bowel gas, and failure to obtain voiding with the catheter in place. Jottrand et al. [20] described active reflux detected by VCUG when spontaneous voiding did not occur during ceVUS. Kennady et al. [32] found that residual urine and microbubble layering could obscure the bladder neck, while mild trabeculation and small diverticula were less conspicuous than with fluoroscopy.

Beyond these technical limitations, several methodological weaknesses of the available evidence should be acknowledged. Many comparative studies have been conducted in relatively small, single-center cohorts, limiting statistical precision and generalizability, although larger observational series have been reported for selected clinical applications. Furthermore, substantial heterogeneity exists in the units of analysis, with outcomes variably reported per patient, examination, kidney–ureter unit, or pelviureteric unit, complicating direct comparisons across studies. The frequent use of VCUG or direct radionuclide cystography as reference standards introduces additional uncertainty, as neither technique can be considered an infallible standard for detecting intermittent reflux. Sequential rather than simultaneous examinations, differences in contrast agents, bladder-filling protocols, the number of filling–voiding cycles, and operator experience further contribute to variability in reported diagnostic performance.

As highlighted by the descriptive methodological comparison in Table 2, additional concerns include potential selection and verification bias, incomplete reporting of blinding and attrition, and inconsistencies in reported numerical results. Importantly, although ceVUS has demonstrated favorable diagnostic performance in several comparative studies, evidence that increased reflux detection translates into improved long-term renal outcomes, reduced antibiotic exposure, or changes in patient management remains limited. These methodological considerations preclude definitive conclusions regarding the superiority, equivalence, or non-inferiority of ceVUS compared with conventional reflux imaging. Accordingly, ceVUS should be regarded as a valuable radiation-free diagnostic option in appropriately selected clinical settings rather than a universally preferred replacement for VCUG.

### 3.8. Radiation-Free Videourodynamics and Other Emerging Applications

The combination of ceVUS with urodynamics extends the technique from reflux detection to simultaneous anatomical and functional assessment. Kapral et al. [30] demonstrated technical feasibility in 25 children, most with myelomeningocele. Microbubbles remained intact after passage through the urodynamic pump and tubing, all examinations were completed, VUR was identified in six patients, and no patient required additional fluoroscopy.

Cvitkovic Roic et al. [31] described a double-lumen-catheter approach that integrates intravesical contrast administration with measurement of intravesical, abdominal, and detrusor pressures. The technique permits documentation of the bladder volume and detrusor pressure at which VUR begins and allows concurrent assessment of IRR. As a technical report, it establishes a workflow rather than validated diagnostic performance.

Kennady et al. [32] subsequently compared ceVUS urodynamics with prior fluoroscopic urodynamics in 75 children, 96% of whom had spinal dysraphism or tethered cord. No significant differences were found in bladder shape, VUR detection, bladder-neck appearance during filling, or urethral appearance during voiding. Continuous ultrasound allowed the precise moment of bladder-neck opening to be observed. The principal limitations were a median interval of 1.3 years between studies, intervening clinical changes, difficulty with mild trabeculation or small diverticula, and microbubble layering in the presence of residual urine.

These studies suggest a future pathway in which children requiring lifelong urodynamic surveillance undergo an initial fluoroscopic study for complete anatomical mapping, followed by ceVUS-based surveillance when the clinical question is functional change, reflux, or bladder-neck behavior. This model is particularly attractive in neurogenic bladder, where repeated radiation exposure begins early and may continue for years.

Other future priorities include standardized agent concentration, physiologic filling endpoints, mandatory two-cycle protocols when clinically tolerated, structured documentation of the phase and volume at reflux onset, and multicenter assessment of the learning curve. The prognostic value of ceVUS-detected IRR should be tested against delayed renal scarring and clinical outcomes. Finally, studies should evaluate whether replacing selected VCUG or acute DMSA examinations with ceVUS reduces radiation, cost, antibiotic exposure, and patient burden without increasing missed clinically important abnormalities.

## 4. Conclusions

CeVUS has matured into a clinically credible, radiation-free examination for pediatric VUR. Comparative evidence shows diagnostic performance broadly comparable to VCUG and direct radionuclide cystography, with particular strengths in continuous observation, repeated cycling, detection of clinically relevant reflux, and visualization of intrarenal reflux. Its capacity to link reflux with parenchymal microbubble extension may add biologic information that is not routinely available from conventional cystography. CeVUS is also increasingly relevant for follow-up, kidney transplantation, and videourodynamics. The technique is well tolerated and strongly accepted by parents. Its principal vulnerabilities include the need for transurethral catheterization, operator dependence and the associated learning curve, limited field of view, potential underdetection of transient grade I reflux, and inconsistent visualization of the distal ureter and ureterovesical junction. Accordingly, ceVUS may be considered a radiation-free option for initial or follow-up assessment in selected pediatric VUR pathways, while VCUG remains appropriate when detailed urethral or complex distal ureteral anatomy must be defined.

Future multicenter studies should prioritize standardized physiologic protocols and patient-centered outcomes rather than simple modality concordance.

## Figures and Tables

**Table 1:** Structured qualitative methodological appraisal of the principal comparative and clinical follow-up studies included in the narrative synthesis. L, low methodological concern; H, high methodological concern; U, unclear methodological concern due to insufficient reporting. Ratings refer to methodological features relevant to interpretation and do not constitute an overall risk-of-bias score. The appropriateness of a comparator reflects its suitability for the stated comparison and does not imply that it constitutes an independent diagnostic gold standard.

Study	Selection	Blinding	Reference	Verfification	Protocol	Study Design and Methodological Characteristics	Methodological Strengths	Main Limitations
Ntoulia et al. [15]	U	U	L	L	H	Prospective, same-day paired ceVUS–VCUG comparison; 30 children and 62 pelviureteric units	Prospective protocol; direct comparison with VCUG; systematic short-term safety monitoring	Small sample; sequential examinations; VCUG used as an imperfect reference standard
Mane et al. [16]	U	H	L	L	H	Comparative study of 30 children and 58 kidney–ureter units; VCUG followed by ceVUS on the same day	Paired examination of individual urinary units; direct comparison of detection and grading	Small sample; sequential testing; ceVUS interpretation by the same sonologist; internal inconsistencies in reported numerical results
Siomou et al. [17]	L	U	L	L	U	Comparative study of 60 infants with mild prenatal hydronephrosis; cyclic ceVUS and VCUG in the same session	Defined clinical population; paired cyclic examinations; assessment of low-grade and higher-grade reflux	No independent reference standard; possible underdetection by VCUG, overclassification by ceVUS, or both; limited generalizability beyond the selected infant population
Kim et al. [18]	L	L	L	L	U	Simultaneous ceVUS–VCUG comparison in 32 children and 63 urinary systems using a combined intravesical contrast mixture	Simultaneous assessment minimizes temporal variability; evaluation of intrarenal reflux; interobserver agreement analysis	Mixed contrast solution differs from routine independent examination protocols; limited sample size
Paltiel et al. [19]	U	U	L	L	H	Retrospective analysis of 101 paired examinations in 97 children; independent blinded ceVUS reassessment	Blinded interpretation by two readers; assessment of reproducibility and diagnostic performance	Retrospective design; sequential examinations; VCUG reports used as reference standard; inconsistency in reported left-sided specificity
Jottrand et al. [20]	U	U	L	L	H	Prospective paired comparison in 89 children and 192 pelviureteric units; contrast viscosity measurements and reader agreement analysis	Prospective design; independent reassessment of ceVUS images; investigation of a potential physical mechanism underlying discordant findings	Sequential rather than simultaneous testing; no independent diagnostic reference standard; viscosity hypothesis not directly tested through controlled manipulation of contrast properties
Viljamaa et al. [21]	U	L	L	L	U	Prospective blinded comparison with direct radionuclide cystography in 22 children; physiologic bladder filling	Independent blinded assessment; standardized physiologic filling volumes; patient- and renal-unit-level analyses	Small sample; no independent definitive reference standard; discordances involving non-dilating reflux
Wang et al. [22]	H	U	L	U	H	Retrospective comparison in 50 infants and 100 pelviureteric units	Paired evaluation of reflux detection and grading	Internal inconsistencies in reported counts and eligibility criteria; bladder filling exceeding twice the predicted capacity, potentially influencing reflux detection
Marschner et al. [24]	H	H	H	H	U	Retrospective single-center clinical cohort of 49 patients; comparison with surgical findings in selected cases	Assessment of ceVUS findings in relation to subsequent clinical management; availability of surgical confirmation in a subgroup	Mixed pediatric and adult population; single-operator interpretation; partial surgical verification with potential selection and verification bias
Tamasi et al. [33]	U	U	H	H	H	Prospective single-center observational cohort of 1050 children; ceVUS-based follow-up strategy for selected patients with VUR	Large clinical cohort; evaluation of follow-up decisions and antibiotic management	No concurrent controlled comparison; inconsistent eligibility, follow-up, and antibiotic discontinuation denominators; limited ability to attribute clinical outcomes specifically to ceVUS

**Table 2:** Principal comparative studies evaluating contrast-enhanced voiding urosonography for pediatric vesicoureteral reflux.

Study	Design/Population	Analysis Denominator	Comparator	Main Findings	Key Methodological Considerations
Ntoulia et al. [15]	Prospective; 30 children, 62 pelviureteric units	62 pelviureteric units	VCUG	Sensitivity 91.7%; specificity 98.0%; agreement 84.3% for detection and 81.8% for grading	Discordances mainly involved low-grade reflux
Mane et al. [16]	Comparative; 30 children, 58 kidney–ureter units	58 kidney–ureter units	VCUG	Sensitivity 100%; specificity 87.8%; PPV 77.3%; NPV 100%; κ = 0.84	Internal numerical inconsistencies in the published report
Siomou et al. [17]	Comparative study in 60 infants with mild prenatal hydronephrosis	Kidney–ureter units	VCUG	ceVUS detected reflux in 18/60 infants vs. 8/60 with VCUG; 25 vs. 12 positive units	No independent reference standard; both techniques performed cyclically
Kim et al. [18]	Simultaneous comparison; 32 children, 63 urinary systems	63 urinary systems	VCUG	ceVUS detected 23/27 refluxing systems vs. 26/27 with VCUG; weighted κ = 0.89	All four ceVUS-missed refluxes were grade I; simultaneous design reduces temporal variability
Paltiel et al. [19]	Retrospective; 101 paired examinations in 97 children	101 paired examinations; kidney-specific analysis	VCUG	Sensitivity 75.0–85.7%; inter-reader κ 0.76–0.78	Blinded independent ceVUS rereading; internal inconsistency in reported left-sided specificity
Jottrand et al. [20]	Prospective; 89 children, 192 pelviureteric units	192 pelviureteric units	VCUG	54 positive units overall; 21 ceVUS-only, 3 VCUG-only, 30 positive with both; ceVUS assigned higher grade in 19/30 concordant positive units	Contrast viscosity proposed as a possible explanation for discordance
Viljamaa et al. [21]	Prospective blinded; 22 children with recurrent UTIs	Patient and renal-unit analyses	Direct radionuclide cystography	Patient-level VUR detection 77.3% with ceVUS vs. 72.7% with radionuclide cystography; renal-unit detection 52.3% vs. 50.0%	Physiologic bladder filling; discordances limited to non-dilating reflux
Wang et al. [22]	Comparative; 50 young infants after UTI	100 pelviureteric units	VCUG	31 positive pelviureteric units with ceVUS vs. 18 with VCUG; κ = 0.547	Conflicting counts across the article and bladder filling > 2× predicted capacity

Abbreviations: ceVUS, contrast-enhanced voiding urosonography; VCUG, voiding cystourethrography; VUR, vesicoureteral reflux; PPV, positive predictive value; NPV, negative predictive value.

## Data Availability

No new data were created or analyzed in this study. Data sharing is not applicable to this article.

## Supplementary Materials

The following supporting information can be downloaded at: https://www.mdpi.com/article/10.3390/diagnostics16213531/s1, Table S1: Database exact search strategy.
