# Supplementary material for: Contrast-Enhanced Voiding Urosonography in Pediatric Vescicoureteral Reflux: A Narrative Review
Source: Diagnostics (Basel). 2026 Oct 27;16(21):3531. doi: 10.3390/diagnostics16213531 (PMC13761738; doi:10.3390/diagnostics16213531)
Supplement: Supplementary file 1 [file diagnostics-16-03531-s001.zip › diagnostics-4602388-supplementary.pdf]

diagnostics

MDPI

Supplementary Materials

Table S1. Database exact search strategy.

Database	Exact search strategy
**PubMed/MEDLINE**	(("contrast-enhanced voiding urosonography"[Title/Abstract] OR "contrast enhanced voiding urosonography"[Title/Abstract] OR ceVUS[Title/Abstract] OR "voiding urosonography"[Title/Abstract] OR "contrast-enhanced cystosonography"[Title/Abstract] OR "contrast enhanced cystosonography"[Title/Abstract]) AND ("vesicoureteral reflux"[Title/Abstract] OR VUR[Title/Abstract] OR "intrarenal reflux"[Title/Abstract]) AND (child*[Title/Abstract] OR pediatric*[Title/Abstract] OR paediatric*[Title/Abstract] OR infant*[Title/Abstract] OR adolescent*[Title/Abstract]))
**Scopus**	TITLE-ABS-KEY(("contrast-enhanced voiding urosonography" OR "contrast enhanced voiding urosonography" OR ceVUS OR "voiding urosonography" OR "contrast-enhanced cystosonography" OR "contrast enhanced cystosonography") AND ("vesicoureteral reflux" OR VUR OR "intrarenal reflux") AND (child* OR pediatric* OR paediatric* OR infant* OR adolescent*))
**Web of Science**	TS=(("contrast-enhanced voiding urosonography" OR "contrast enhanced voiding urosonography" OR ceVUS OR "voiding urosonography" OR "contrast-enhanced cystosonography" OR "contrast enhanced cystosonography") AND ("vesicoureteral reflux" OR VUR OR "intrarenal reflux") AND (child* OR pediatric* OR paediatric* OR infant* OR adolescent*))
